# Clinical outcome of Two Minute Walk Test after return to run program in IDEO users: a retrospective study

**DOI:** 10.3389/fresc.2025.1602110

**Published:** 2025-10-08

**Authors:** Michelle D. Lockwood, Christopher F. Hovorka, Molly E. Baumann, W. Lee Childers

**Affiliations:** ^1^ Research and Surveillance Division, Extremity Trauma and Amputation Center of Excellence, Falls Church, VA, United States; ^2^ Defense Health Agency, Falls Church, VA, United States; ^3^ Department of Rehabilitation Medicine, Center for the Intrepid, Brooke Army Medical Center, San Antonio, TX, United States; ^4^ Oak Ridge Institute for Science and Education, Oak Ridge, TN, United States

**Keywords:** Intrepid Dynamic Exoskeletal Orthosis (IDEO), clinical outcome measure, Two Minute Walk Test (2MWT), lower limb musculoskeletal injuries (MSKIs), functional mobility

## Abstract

**Objective:**

To investigate clinical improvement in persons with severe musculoskeletal injuries following a 4-week high intensity sports-based return to run rehabilitation therapy program (RTR) combined with use of an Intrepid Dynamic Exoskeletal Orthosis (IDEO).

**Patients:**

41 persons (37 male and 4 female) with lower limb musculoskeletal injuries undergoing treatment and rehabilitation at the Center for the Intrepid were included in the retrospective cohort study.

**Methods:**

Retrospective analysis of clinical data was used to identify changes in Two Minute Walk Test (2MWT) outcomes in patients with lower limb musculoskeletal injuries without the IDEO, with the IDEO alone and after IDEO + RTR. A repeated measures ANOVA was used to examine differences in distance walked across the three groups (NO IDEO, IDEO alone, and IDEO + RTR). Bonferroni *post-hoc* analysis was performed, and a level of significance was set at 0.05.

**Results:**

A statistically significant difference in distance walked between all groups was observed (*p* < 0.001). Patients' mean distance walked using the IDEO alone (179 m, *p* < 0.001) and IDEO + RTR (208 m, *p* < 0.001) increased significantly compared to NO IDEO (152 m, *p* < 0.001). These differences surpass the Minimal Clinically Important Difference (37.2 m) and the Minimal Detectable Change (34.3 m) for the 2MWT in the limb loss population.

**Conclusion:**

These data suggest the potential benefit of the combination of IDEO + RTR improved walking in patients with lower limb musculoskeletal injuries and suggest the 2MWT may be a meaningful, simple measure to detect improvement in function.

## Introduction

**Figure 1:**
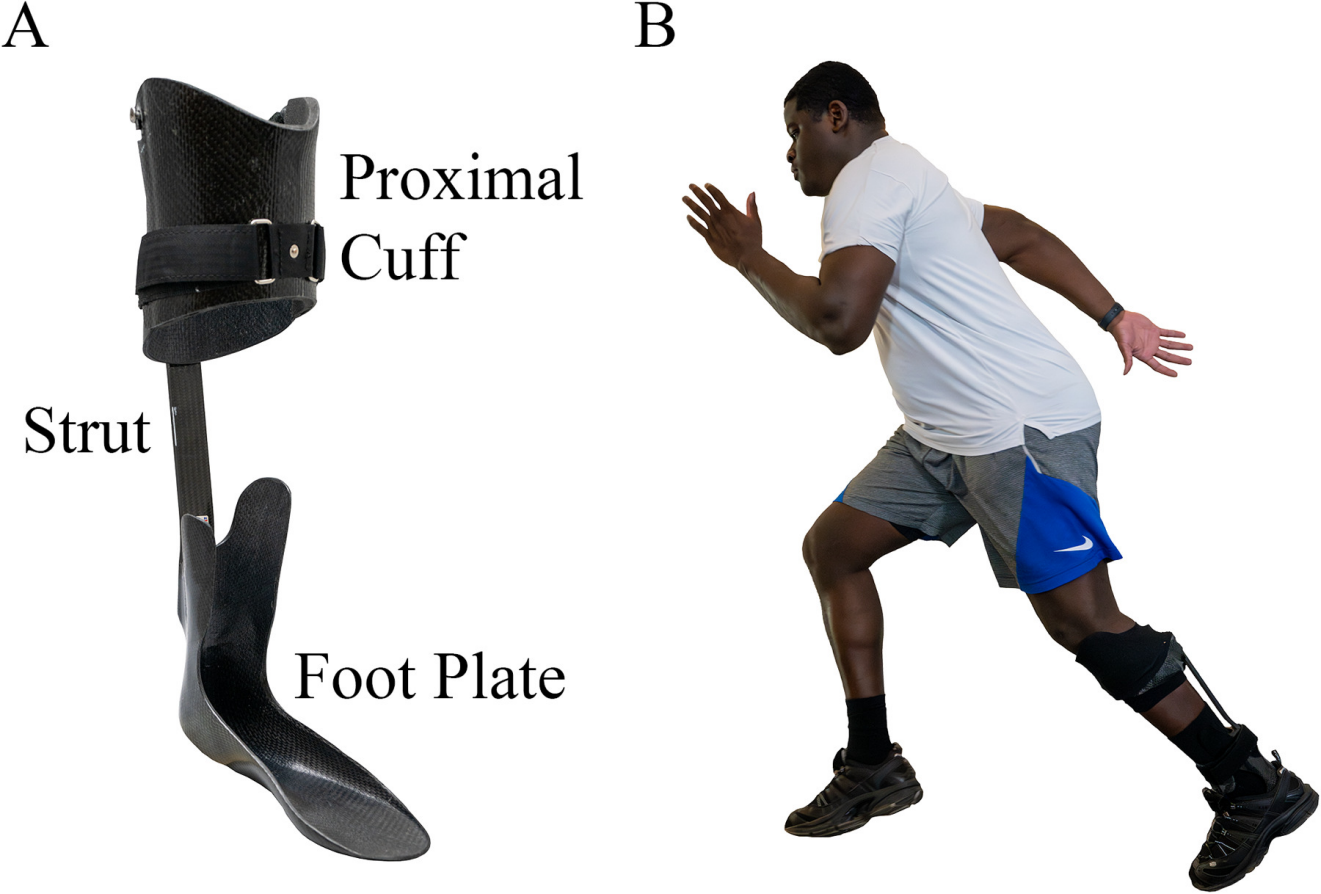
**(A)** The IDEO provides rigid custom molded support of the affected lower extremity by incorporating a carbon composite adjustable shank cuff and ankle-foot sections joined by a carbon composite flexible spring strut anchoring the posterior aspect of the shank and ankle-foot sections to enable energy storage and return. **(B)** Patient wearing the IDEO undergoing activity training in the IDEO + RTR program.

Severe lower extremity musculoskeletal injuries (MSKIs) often result in chronic functional impairments that limit a person's participation in rigorous physical activities (1, 2). These individuals also face decisions of limb amputation due to severe pain and loss of mobility (3–6). One treatment strategy to enable Service members (SMs) with lower limb MSKIs to return to active duty is to fit them with the Intrepid Dynamic Exoskeletal Orthosis (IDEO) (Figure 1). Additionally, these SMs participate in the specifically developed return to run program (RTR) for IDEO users (4, 5).

Orthoses are commonly used in gait rehabilitation to improve walking mechanics, balance, and mobility in individuals with lower limb impairments. Physical therapy complements orthotic treatment by prescribing exercises to enhance strength, flexibility, agility, balance, and coordination, addressing both mechanical and neuromuscular aspects of gait. Recent advances in orthotic technology include two carbon fiber ankle foot orthoses similar to the highly-specialized, custom-made IDEO. Unlike the IDEO, these orthoses are available to civilians. The ExoSym, like the IDEO, is prescribed to reduce pain and allow patients to regain functional mobility and return to high-impact activities after experiencing severe lower limb injuries where amputation might be strongly considered (7). The ExoSym is a lighter, slimmer version of the IDEO. Like the IDEO, the ExoSym has an associated rehabilitation program. The ExoSym rehabilitation program is a less rigorous regimen consisting of 5- to 7-days of techniques training compared to the 4-week intensive IDEO RTR program (7). The ReAktiv Posterior Dynamic Strut Element (PDE^TM^) is another energy storing orthosis that is also paired with an undescribed rehabilitation program (8). The ReAktiv PDE^TM^ differs from the IDEO and ExoSym in that it has an integrated modular strut system that can be adjusted by the patient and clinician based on height, weight, activity level, and degree of unloading that is needed to meet specific patient needs (8).

To determine treatment efficacy, the two-minute walk test (2MWT) can be used to detect patients' function before, during, and after their treatment. The 2MWT is a valid and reliable clinical outcome measure often used to quantify patients’ overall health status and functional capacity post-stroke (9–12). The 2MWT remains understudied in MSKIs. Given its simplicity and minimal instrumentation required for implementation (e.g., measuring distance ambulated within two minutes) it affords itself high utility (9, 10). Furthermore, 2MWT results provide clinicians information for quantifying the therapeutic response to treatment and help inform treatment decision making (12, 13).

A goal of the IDEO + RTR program is to return service members to high-level function with an increased likelihood of returning to active duty (14). The RTR program is a four-week high-intensity sports-like, team-oriented rehabilitation program that includes physical and behavioral therapies designed specifically for persons that use the IDEO (15). The RTR program teaches movement strategies that maximize mechanical energy storage and return capabilities of the IDEO (15). How to properly load and utilize the IDEO is a focus of the RTR program in addition to strengthening and multidirectional agility training (15).

As evidence-based healthcare seeks to quantify and validate healthcare intervention, strategic use of measurements that are clinically relevant, simple, inexpensive, reliable, and reproducible are increasingly important to substantiate treatment interventions in a busy clinical practice setting. Though the 2MWT has been utilized for a variety of medical conditions and populations, its use to evaluate persons with lower limb MSKIs have been limited. Therefore, the purpose of our investigation was to examine 2MWT scores before and after the combination of IDEO + RTR. The 2MWT is a clinical outcome measure routinely collected in our clinic and provides insight into patients' functionality for this retrospective analysis. We hypothesize we will detect meaningful improvement in walking distance with the 2MWT in individuals with lower limb MSKIs after IDEO + RTR.

## Materials and methods

### Ethical approval

This study protocol was reviewed and deemed not research by the San Antonio Institutional Review Board in compliance with all applicable Federal regulations governing the protection of human subjects. Written approval was obtained for use of identifiable photographs.

### Study design

This study was a retrospective cohort design to evaluate a clinical program.

### Participants

Subject demographics, mechanism of injury, and 2MWT scores were extracted from medical records. Data was extracted and included in the analysis for any individual who received an IDEO and completed the RTR program with 2MWT scores for all three timepoints of interest (during casting for the IDEO prior to provision, at IDEO provision, after completion of the 4-week RTR program). As part of the program, no additional assistive devices are allowed except for the IDEO.

### Intervention

The intervention in this study consisted of the IDEO and the RTR program. Participants in RTR were trained daily during the week by a physical therapist on how to use the orthosis to increase the speed of ambulation from walking to running. In the RTR program, individuals are taught how to apply force correctly to the footplate to optimize the bending of the strut that returns the energy during push-off. Subjects participate in prescribed activities to gain strength, agility, and endurance, facilitating a return to high activity. By utilizing the IDEO during participation in RTR, the clinical goal is to increase subjects' function during limb salvage. The end goal is to prevent amputation by reducing pain and providing support to weakened musculature.

### Outcome measures

Clinicians collected the 2MWT at clinically relevant timepoints as part of standard care. This outcome measure is simple and straightforward to collect during a clinical appointment and is part of a routine assessment of treatment outcome at CFI for patients using the IDEO. The timepoints utilized for this retrospective study were the day of evaluation and casting for an IDEO device (NO IDEO condition), after evaluating and adjusting the IDEO for fit, comfort and safety on the day of provision (IDEO condition), and at completion of the 4-week RTR program (IDEO + RTR condition). There is around a 4-week gap between casting and IDEO provision. Patients start the RTR program within a week of receiving the IDEO.

### Data collection and analysis

For the 2MWT, patients were instructed to walk as quickly as possible on a 60 m indoor track for 2 min while time and distance were recorded in each patients' clinical notes. This 2MWT data along with the patient's age, sex, injury mechanism, race, height, and weight were extracted from the medical chart for retrospective analysis.

To determine differences between the mean walking distance of patients in each group, a repeated measures ANOVA was used. If statistical differences were observed between time points, a Bonferroni *post-hoc* analysis was conducted. Mauchly's test of sphericity was utilized to evaluate the assumption of sphericity. Effect size $\left(\eta_{p}^{2}\right)$ represents the influence the independent variable (utilizing the IDEO + RTR program) has on the dependent variable (distance walked). An effect size of 0.01 will be considered small, 0.06 medium, and values above 0.14 will be considered large. Significance was set to *p* < 0.05. Group means (standard deviation) are reported for walking distance. Comparison of 2MWT results to Minimally Detectible Change (MDC) and Minimal Clinically Important Difference (MCID) of persons with limb loss was made (nearest population available).

## Results

**Figure 2:**
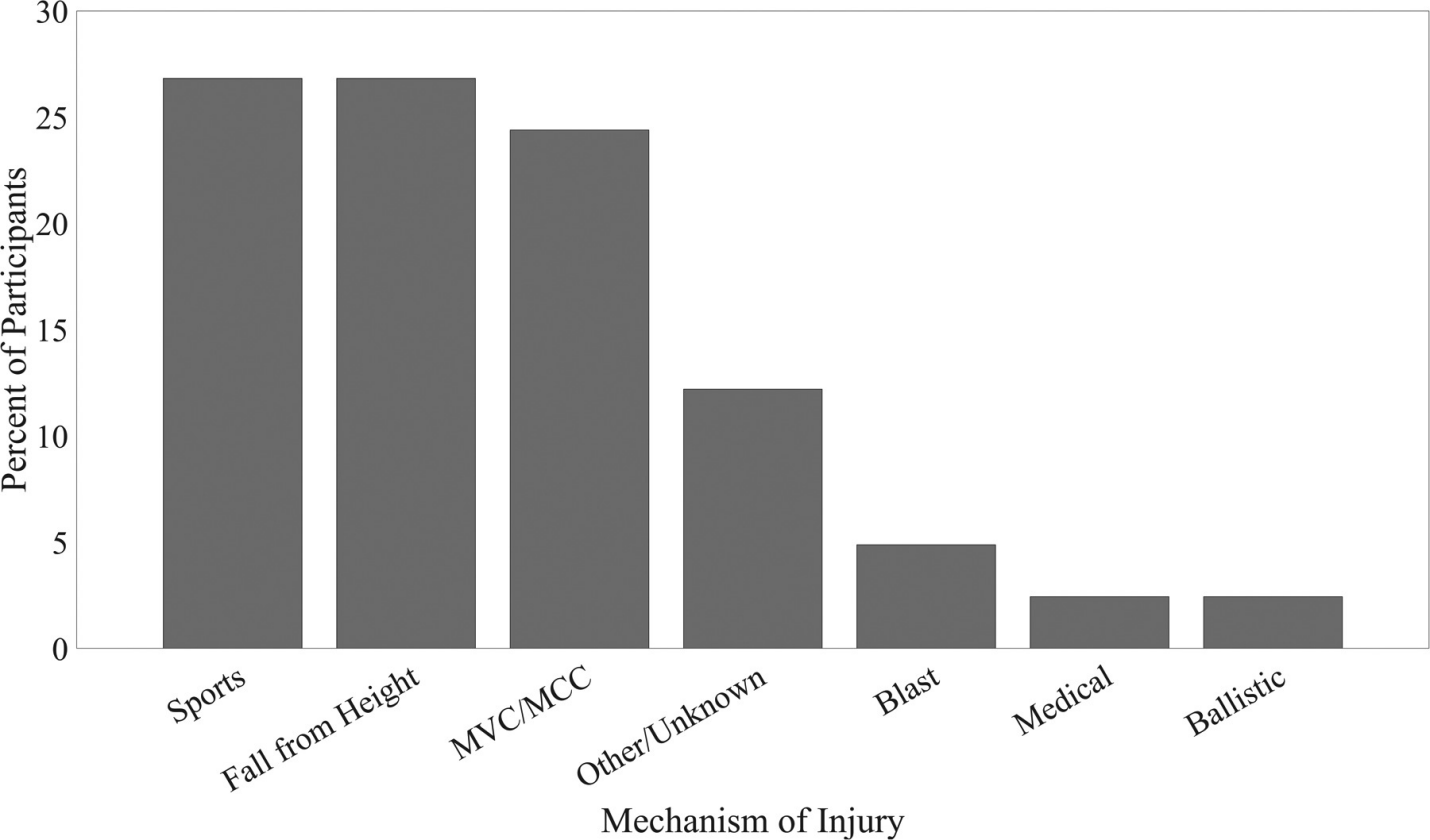
Mechanism of injury. Patients most frequently reported sports, falls, and motor vehicle crash (MVC) or motorcycle crash (MCC) as their mechanism of injury.

Forty-one persons, consisting of 37 male and 4 female; mean age 36.6 (8.2) years were included in the study. For detailed demographic information, see Table 1. The mechanism of injury was categorized as primarily trauma to the lower extremity (Figure 2).

**Table 1:** Demographic characteristics (Mean ± SD).

Characteristics	*n*	%
Sex, *n* (%)		
Female	4	9.8%
Male	37	90.2%
Patient metrics		
Age (years)	36.6 ± 8.4	
Age (range)	20–54	
Height (cm)	177.0 ± 8.0	
Mass (kg)	91.5 ± 15.3	
Race		
Caucasian	33	80.5%
Pacific Islander	4	9.8%
Black/African American	3	7.3%
Other	1	2.4%

**Figure 3:**
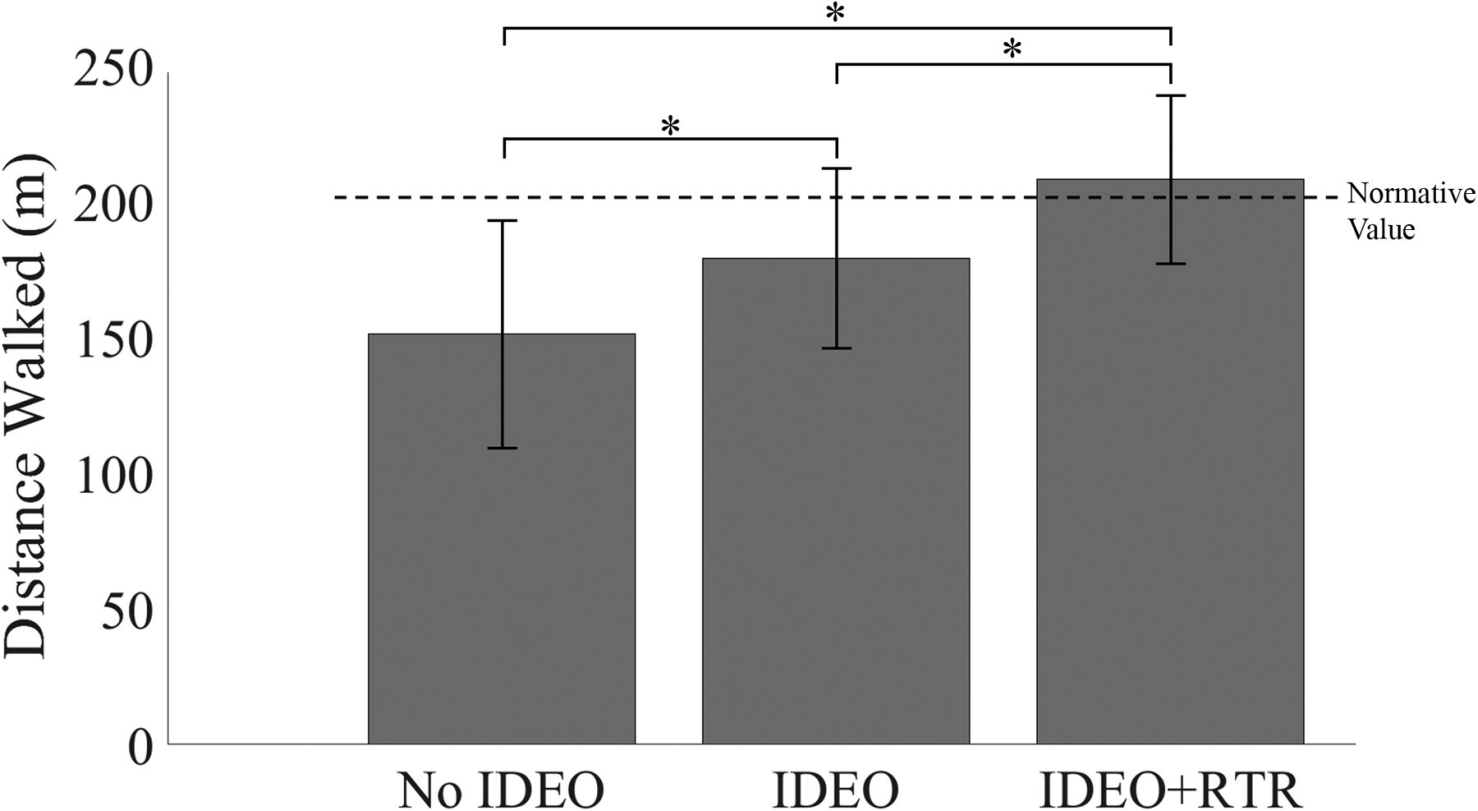
The mean (SD) 2MWT distance walked increased significantly in patients using the IDEO alone (*p < 0.001;) and with the combined IDEO + RTR treatment intervention (*p < 0.001) compared to the no IDEO group. The dashed line (202.1 m) represents the distance walked for healthy age matched individuals (30–40 yrs.) (16). Comparison of the IDEO and IDEO + RTR study results to the normalized value demonstrates an overall return to a healthy walking distance as a group only in the combined IDEO + RTR therapeutic intervention. Significance: *p < 0.001, *n* = 41.

Mauchly's test for sphericity had not been violated for distance [*χ*^2^ (2) = 5.775, *p* = 0.056]. A statistically significant difference in distance walked between all groups was observed (*F*_(2,80)_ *=* 95.900, *p* < 0.001, $\eta_{p}^{2}$ = 0.706) (Figure 3). Walking distance after IDEO + RTR treatment [208 (29) m], 95% CI 198, 217) increased 37% compared to the NO IDEO group [152 (40) m, 95% CI 139, 164]. Walking distance after IDEO treatment alone [179 (31) m, 95% CI 170, 189] increased 18% compared to the NO IDEO group (*p* < 0.001). There was a 16% increase in distance walked after patients completed IDEO + RTR training compared to IDEO alone (*p* < 0.001). Overall, 61% (*n* = 25, 95% CI 19, 32) of patients returned to a healthy walking distance as reported in the literature following IDEO + RTR treatment that is noted by the dashed line in Figure 3 (16). Twenty-four percent (*n* = 10) of patients with the IDEO only condition walked to the normative distance value. No patients without the IDEO were above the normative walking distance. These 2MWT improvements with the IDEO + RTR compared to NO IDEO (56 m), exceed both the MDC (34.3 m) and MDIC (37.2 m) of patients with limb loss.

## Discussion

The results from the 2MWT suggest the effectiveness of the IDEO + RTR approach in enhancing the functional abilities of individuals with lower limb MSKIs, with notable increases in walking performance attributed to the inclusion of the RTR program. Accordingly, the main finding of this study is that patients showed significant improvements in distance walked during the 2MWT after undergoing a combination of IDEO and RTR treatments. The 2MWT is a straightforward outcome measure for clinicians to administer and yields accurate, repeatable results. This provides the ability to monitor treatment responses in individuals with lower limb MSKIs.

The 2MWT reflects an aggregate of integrated body systems functions (e.g., cardiovascular, neuromuscular, skeletal, etc.) that contribute to balance, stability, and physical capability (9, 17). Integrated body systems functions are critical because they contribute to the overall functional mobility required for individuals to participate in rigorous physical activity.

Significant improvements in patients' walking distance found in this study suggest the use of IDEO + RTR treatment may increase the functional ability of individuals with lower limb MSKIs. IDEO + RTR provided enough improvement to return patients to their healthy age-matched cohort for walking distance with the 2MWT. This demonstrates the capability of the 2MWT as an assessment measure to detect enhanced outcomes associated with combination therapies such as IDEO + RTR.

These findings align with previous reports indicating that individuals with foot drop experienced improved walking speeds when receiving the IDEO + RTR combination treatment, walking farther and faster compared to the IDEO alone. In this same study, the combination of IDEO + RTR treatment significantly reduced the time to complete the 10 m Walk Test by 2.3 s (18). While our study utilized the 2MWT rather than the 10 m Walk Test, the increased distance walked by subjects during the 2MWT means the average walking speed was increased in our study as well. In another study, subjects using the commercially available, IDEO-like, ReAktiv PDE™ orthosis to increase walking speed that surpassed the MCID for persons with limb loss and helped patients achieve normative walking levels for age-matched persons without injury (8).

The 2MWT not only differentiates function between treatment groups, but can also track individual subject's progress. In a similar study, participants achieved a 42% increase in walking speed compared to no treatment intervention, consistent with age-matched persons without injury (8). Similarly, we report in the present study that 61% of individuals achieved an age-matched healthy walking speed after IDEO + RTR. Persons that remained below the normative reference value following IDEO + RTR exhibited a mean 36% increase in walking distance compared to the IDEO alone. These same individuals continued to improve with an additional increase in walking distance of 15% following IDEO + RTR treatment. Rehabilitation is an individualized process and this data suggests that the rate of improvement may not be consistent between individuals. Hence, the rate of change of walking distance may offer additional perspective on patient's improvement in functional status during treatment. Future work may look at how the rate of change impacts overall outcomes.

The MDC and MCID for persons with lower limb loss demonstrated the clinical significance of findings in this study. Improvement in walking distance after the combination of IDEO + RTR (56 m), patients exceeded both the MDC (34.3 m) and MCID value (37.2 m) when compared to NO IDEO (19, 20). Though a statistically significant increase in mean walking distance was identified, participants using IDEO treatment alone did not surpass the MCD, MCID, or reach healthy normative values. This finding underscores the added value of physical therapy in combination with use of an orthosis (19, 20). The combination of IDEO + RTR is the recommended treatment for individuals at the CFI with lower limb MSKIs because it enabled patients to exceed the desired functional threshold.

This integrated approach of combining the use of orthoses with intensive, function-based rehabilitation (IDEO + RTR) aligns with current best-practices for rehabilitation of lower limb MSKIs (8, 21–24). This combined treatment approach with a highly durable carbon fiber energy storing ankle foot orthosis has proved beneficial to patients. The orthosis provides support, pain reduction, and enables mobility while high-intensity rehabilitation exercise programs provide additional gains in walking distance, function, and patient outcomes (25). Functional gains observed in the 2MWT highlight this improvement in the combinatorial approach to rehabilitation. These functional gains could potentially be explained by two principal mechanisms: (1) spring dynamics of the IDEO and, (2) conditioning of the participants' limb strategies to enhance lower limb propulsion. Design of the IDEO exploits a principle of spring dynamics by incorporating a composite posterior leaf spring. Persons using the IDEO while engaging in high energy activities impose notable bending moments on their lower limbs and IDEO strut which increases mechanical energy. The RTR program trains participants to intentionally load the footplate of the IDEO and coordinate hip and knee strategies that optimize IDEO strut bending-unbending cycles and energetics. These limb coordination strategies are incorporated into task practice such as running with progressive increases in intensity and duration that enables a training effect (e.g., motor recruitment, neuromuscular adaptation and coordination of new and updated limb strategies) to enhance lower limb propulsion.

### Study limitations

There were several limitations to this study. As a retrospective study, there was no true control group of individuals that received an IDEO, without RTR. This is the clinical standard at CFI and IDEOs are not provided as a treatment unless the patient commits to participating in the RTR program to ensure appropriate use of the orthosis. A comparison group of time in IDEO without specialized training would better inform the full benefit of the RTR program. This could potentially be more closely realized by partnering with another institution that does not have a RTR program, but utilizes an IDEO-like device. There is a possibility of a learning effect for the 2MWT. Patients may have more familiarity and comfort with the 2MWT having completed it before. They may have also been actively attempting to exceed their previous distance which could confound the results. The short time frame of follow-up is a limitation. Longer follow-up periods to evaluate if the benefit is maintained after discharge from the treatment program at the CFI would be beneficial.

### Future works

Future research should aim to help define the influence of the treatment modality (e.g., the IDEO and the combination of IDEO + RTR) individually and together. The optimal treatment dosage (frequency, intensity, duration of RTR program) needs further study to understand if improvement is continuous or if there is a stabilized level. This strategy can better inform planning and implementation of these interventions to enhance clinical practices and improve patient outcomes.

## Conclusion

Results from this study enhance understanding of functional mobility recovery using the 2MWT which is a simple, yet effective, clinical measurement tool that accurately represents a person's functional capacity. This study highlights the significant benefits of the combination of IDEO + RTR treatments for individuals with lower limb MSKIs, as evidenced by the improvements in walking distance measured by the 2MWT. Subjects' walking distance during the 2MWT significantly improved through each phase of rehabilitation from IDEO provision to completion of the IDEO + RTR program. This suggests the benefit of combinatorial treatments to treat lower limb MSKIs to improve subjects' walking performance.

## Data Availability

The original contributions presented in the study are included in the article/Supplementary Material, further inquiries can be directed to the corresponding author.

## References

[1] Butowicz CM Dearth CL Hendershot BD. Impact of traumatic lower extremity injuries beyond acute care: movement-based considerations for resultant Longer term secondary health conditions. Adv Wound Care (New Rochelle). (2017) 6(8):269–78. 10.1089/wound.2016.0714 28831330 PMC5564032

[2] Butcher JL MacKenzie EJ Cushing B Jurkovich G Morris J Burgess A Long-term outcomes after lower extremity trauma. J Trauma. (1996) 41(1):4–9. 10.1097/00005373-199607000-00002 8676422

[3] Dirschl DR Dahners LE. The mangled extremity: when should it be amputated? J Am Acad Orthop Surg. (1996) 4(4):182–90. 10.5435/00124635-199607000-00002 10795053

[4] Patzkowski JC Blanck RV Owens JG Wilken JM Blair JA Hsu JR. Can an ankle-foot orthosis change hearts and minds? J Surg Orthop Adv. (2011) 20(1):8–18. 21477527

[5] Hoyt B Nelson S Fay J Wade S Brooks DI Potter B. IDEO energy-storing orthosis: effects on lower extremity function and preservation. Injury. (2021) 52:3505–10. 10.1016/j.injury.2021.07.023 34311958

[6] Farrelly E Tarapore R Lindsey S Wieland MD. Management of the mangled extremity. Surg Clin North Am. (2024) 104(2):385–404. 10.1016/j.suc.2023.10.006 38453309

[7] Williamson JN Grunst MM Lynn J Williamson GA Blanck RV Wilken JM. Short-term effect of a carbon fiber custom dynamic orthosis and integrated rehabilitation on self-reported physical function, pain, speed, and agility in civilians. Prosthet Orthot Int. (2023) 47(6):607–13. 10.1097/PXR.0000000000000252 38064296

[8] Gardner S Frecklington M Rose K Carroll MR. Changes in functional outcomes in people with high-energy ankle trauma after the use of the ReAktiv posterior dynamic element orthosis and a rehabilitation program: a case series. Prosthet Orthot Int. (2024) 48(4):368–71. 10.1097/PXR.0000000000000291 39140760 PMC11323753

[9] Fritz S Lusardi M. White paper: “walking speed: the sixth vital sign”. J Geriatr Phys Ther. (2009) 32(2):46–9. 10.1519/00139143-200932020-00002 20039582

[10] Middleton A Fritz SL Lusardi M. Walking speed: the functional vital sign. J Aging Phys Act. (2015) 23(2):314–22. 10.1123/japa.2013-0236 24812254 PMC4254896

[11] Studenski S Perera S Patel K Rosano C Faulkner K Inzitari M Gait speed and survival in older adults. JAMA. (2011) 305(1):50–8. 10.1001/jama.2010.1923 21205966 PMC3080184

[12] Cesari M Kritchevsky SB Penninx BW Nicklas BJ Simonsick EM Newman AB Prognostic value of usual gait speed in well-functioning older people–results from the health, aging and body composition study. J Am Geriatr Soc. (2005) 53(10):1675–80. 10.1111/j.1532-5415.2005.53501.x 16181165

[13] Montero-Odasso M Schapira M Soriano ER Varela M Kaplan R Camera LA Gait velocity as a single predictor of adverse events in healthy seniors aged 75 years and older. J Gerontol A Biol Sci Med Sci. (2005) 60(10):1304–9. 10.1093/gerona/60.10.1304 16282564

[14] Blair JA Patzkowski JC Blanck RV Owens JG Hsu JR, Skeletal Trauma Research C. Return to duty after integrated orthotic and rehabilitation initiative. J Orthop Trauma. (2014) 28(4):e70–4. 10.1097/BOT.0000000000000006 24121984

[15] Bedigrew KM Patzkowski JC Wilken JM Owens JG Blanck RV Stinner DJ Can an integrated orthotic and rehabilitation program decrease pain and improve function after lower extremity trauma? Clin Orthop Relat Res. (2014) 472(10):3017–25. 10.1007/s11999-014-3609-7 24744130 PMC4160498

[16] Bohannon RW. Normative reference values for the two-minute walk test derived by meta-analysis. J Phys Ther Sci. (2017) 29(12):2224–7. 10.1589/jpts.29.2224 29643611 PMC5890237

[17] Rasmussen LJH Caspi A Ambler A Broadbent JM Cohen HJ d'Arbeloff T Association of neurocognitive and physical function with gait speed in midlife. JAMA Netw Open. (2019) 2(10):e1913123. 10.1001/jamanetworkopen.2019.13123 31603488 PMC6804027

[18] Franklin N Hsu JR Wilken J McMenemy L Ramasamy A Stinner DJ. Advanced functional bracing in lower extremity trauma: bracing to improve function. Sports Med Arthrosc Rev. (2019) 27(3):107–11. 10.1097/JSA.0000000000000259 31361720

[19] Resnik L Borgia M. Reliability of outcome measures for people with lower-limb amputations: distinguishing true change from statistical error. Phys Ther. (2011) 91(4):555–65. 10.2522/ptj.20100287 21310896

[20] Carse B Scott H Davie-Smith F Brady L Colvin J. Minimal clinically important difference in walking velocity, gait profile score and two minute walk test for individuals with lower limb amputation. Gait Posture. (2021) 88:221–4. 10.1016/j.gaitpost.2021.06.001 34119776

[21] Crowell MS Deyle GD Owens J Gill NW. Manual physical therapy combined with high-intensity functional rehabilitation for severe lower extremity musculoskeletal injuries: a case series. J Man Manip Ther. (2016) 24(1):34–44. 10.1179/2042618614Y.0000000076 27252581 PMC4870039

[22] Anderson KM McGregor AH Masouros SD Wilken JM. Orthotics. In: Bull AMJ Clasper J Mahoney PF, editors. Blast Injury Science and Engineering: A Guide for Clinicians and Researchers. Cham: Springer International Publishing (2022). p. 437–46.

[23] Jorge M. Orthotics and prosthetics in rehabilitation: multidisciplinary approach. In: Chui KK, Jorge M, Yen SC, Lusardi MM, editors. *Orthotics and Prosthetics in Rehabilitation*. 4th ed. St. Louis, MO: Elsevier (2020). p. 2–13. 10.1016/B978-0-323-60913-5.00001-5

[24] You DZ Leighton JL Schneider PS. Current concepts in rehabilitation protocols to optimize patient function following musculoskeletal trauma. Injury. (2020) 51(Suppl 2):S5–9. 10.1016/j.injury.2020.03.047 32418645

[25] Grunst MM Wiederien RC Wilken JM. Carbon fiber ankle-foot orthoses in impaired populations: a systematic review. Prosthet Orthot Int. (2023) 47(5):457–65. 10.1097/PXR.0000000000000217 36779973 PMC12345332

